# Nickel-resistant gut microbiota: a missing link between environmental exposure and metabolic disease

**DOI:** 10.3389/fmicb.2026.1852314

**Published:** 2026-07-03

**Authors:** Elena Angela Lusi, Claudia Rifici

**Affiliations:** 1 St Vincent Health Care Group-UCD, Dublin, Ireland; 2 Department of Veterinary Science, University of Messina, Messina, Italy

**Keywords:** gut microbiome, intestinal microbiota, metabolic syndrome, nickel allergy, obesity, overweight

## Abstract

Environmental factors are emerging as important modulators of the gut microbiome, with significant implications for metabolic health. Among these, nickel—a ubiquitous dietary metal traditionally regarded as an allergen—is gaining recognition as a systemic immune-metabolic modulator. Chronic nickel exposure has been linked to overweight and metabolic alterations, particularly in nickel-allergic individuals, suggesting that environmental nickel may represent an underrecognized contributor to metabolic dysfunction in susceptible populations. Recent studies have identified nickel-resistant bacteria within the gut microbiota of affected individuals, providing a biologically plausible framework linking environmental metal exposure to microbial ecology. These microorganisms contribute to nickel detoxification and may influence host physiology through interactions with microbial metabolism, energy balance, and immune signaling. Under conditions of chronic exposure, ecological selection of nickel-resistant communities may contribute to dysbiosis and altered host–microbiome interactions. By integrating clinical observations with emerging microbiological evidence, this Perspective explores the hypothesis that nickel-resistant gut microbiota may represent candidate mediators at the intersection of environmental exposure, immunity, and metabolism. Understanding how dietary metals shape microbial ecosystems may provide new insights into metabolic disease and highlights metal–microbiota interactions as a promising area for future investigation.

## Introduction

The human gut microbiota is a central regulator of host metabolism, immune homeostasis, and systemic physiology, acting as a dynamic interface between environmental exposures and health outcomes. However, identifying specific environmental drivers capable of linking immune responses, microbial ecology, and metabolic disease remains a major challenge.

A pivotal advance in this field was the demonstration that chronic dietary nickel exposure is associated with overweight in women, particularly in those with nickel allergy (Lusi et al., 2015). This finding provided the first evidence supporting a relationship between an environmental metal, immune reactivity, and metabolic phenotype, suggesting that nickel may contribute to obesity beyond its classical role as a contact allergen. Subsequent studies, including a large cohort analysis, confirmed that nickel-allergic individuals exhibit higher body mass index and worse metabolic profiles compared to non-allergic controls (Watanabe et al., 2018).

These observations support a paradigm shift in which nickel is no longer viewed solely as an allergen but rather as a systemic immune-metabolic modulator capable of influencing metabolic homeostasis, particularly in susceptible populations. In line with this, nickel exposure has been linked to low-grade chronic inflammation, a recognized driver of obesity and metabolic syndrome (Zambelli et al., 2016; Rehman et al., 2018).

The later isolation of nickel-resistant bacteria from the gut microbiota of obese, nickel-allergic women (Lusi et al., 2017) provided a microbiological framework, revealing that elevated dietary nickel acts as a strong ecological selector, favoring microbial populations capable of surviving extremely high nickel concentrations. This work opened a novel avenue for understanding metal–microbiota interactions, highlighting the nickel-gut microbiome axis as a previously unrecognized potential contributor to metabolic dysregulation.

Why nickel rather than other metals? Nickel occupies a unique intersection of widespread dietary exposure, potent immunogenicity, and demonstrated impact on metabolism and microbiota composition. While metals such as cadmium, lead, or copper also interact with host physiology and microbiota, few combine high prevalence in the diet, immune activation, and evidence of directly selecting resistant gut microbes. Nickel thus represents a powerful model to explore how environmental metals act as ecological selectors and immune-metabolic modulators, providing mechanistic insights into the gut microbiome as a missing link in metabolic dysregulation (Bruins et al., 2000; Nies, 2003; Rehman et al., 2018).

**Figure 1:**
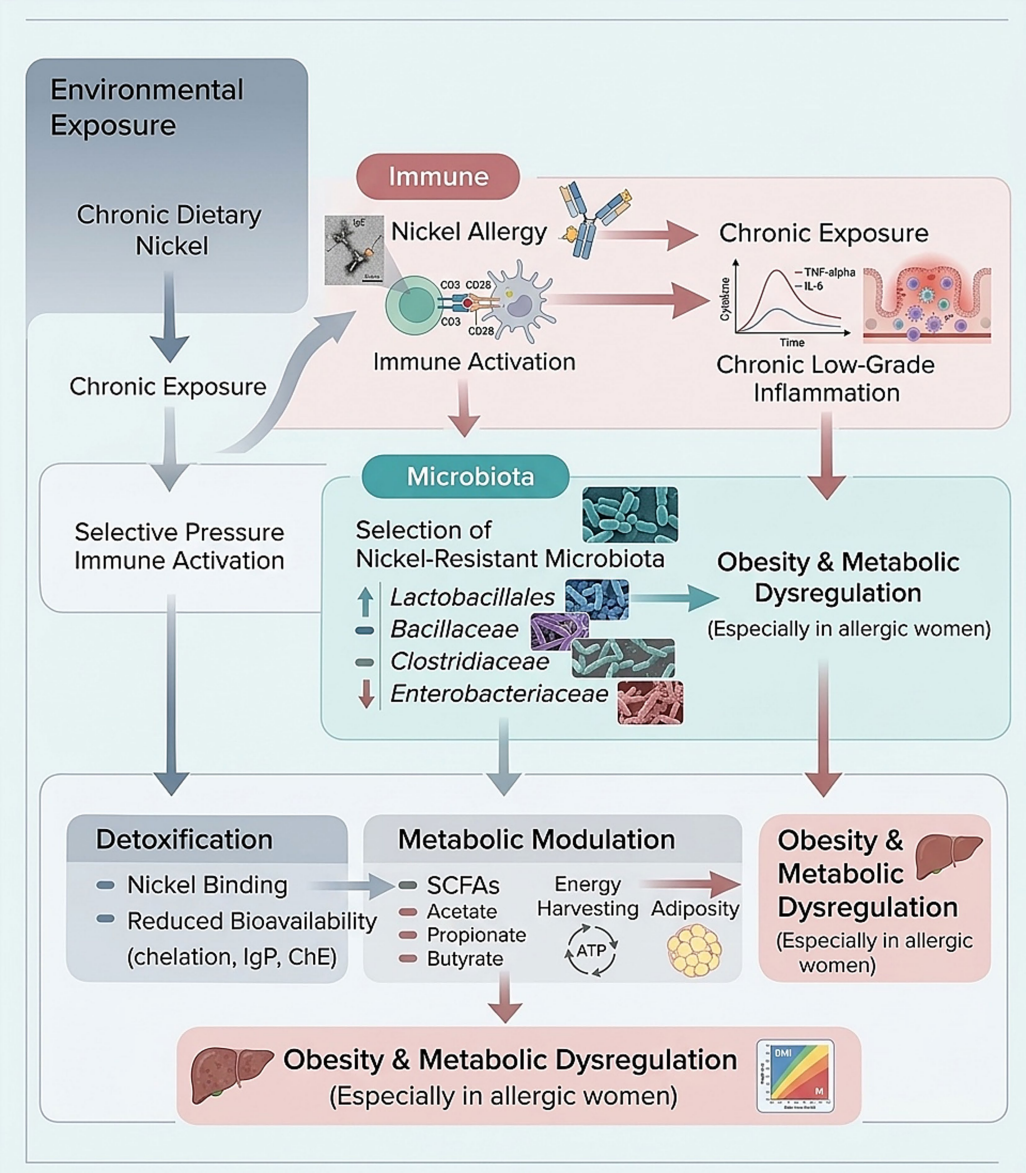
Nickel as a multilevel driver of immune-metabolic and microbiota-mediated effects. Chronic dietary nickel acts simultaneously as an environmental stressor, immune modulator, and ecological selector. In susceptible individuals, particularly women with nickel allergy, it promotes low-grade systemic inflammation and immune activation while selectively enriching nickel-resistant gut taxa—including *Lactobacillales*, *Bacillaceae*, and *Clostridiaceae*—and reducing Enterobacteriaceae. These microbes form the nickel-resistant gut microbiome, a previously underrecognized candidate biological interface linking environmental metal exposure to metabolic disruption. By detoxifying nickel and potentially influencing host metabolism and immune signaling, these bacteria occupy a dual role: protective under acute exposure but potentially maladaptive under chronic exposure, contributing to dysbiosis, increased energy harvest, adiposity, and metabolic syndrome. This integrated framework positions metal–microbiota interactions as a plausible biological framework connecting environmental exposure, microbial ecology, and metabolic disease across human and animal systems.

Taken together, these findings suggest that nickel exerts multilevel biological effects: as an allergen, a metabolic modulator, and a selective force shaping gut microbiota composition and function. This integrated perspective sets the stage for exploring the nickel-gut microbiome axis as a critical missing link between environmental exposure and metabolic disease (Figure 1).

## The impact of nickel on gut microbiota composition and function

One of the most intriguing developments in this field has been the identification of nickel-resistant bacteria within the human gut microbiota. The discovery of these microorganisms provided a biological framework through which chronic dietary nickel exposure may influence host–microbiome interactions beyond its established role as an allergen. Microbial communities isolated from nickel-allergic overweight individuals were found to tolerate nickel concentrations far exceeding those observed in non-allergic controls, suggesting long-term ecological adaptation to metal exposure (Lusi et al., 2017).

The available evidence indicates that nickel acts as a powerful ecological selector, favoring the expansion of microbial populations capable of surviving under elevated metal concentrations. Similar adaptive responses have been extensively described in environmental microbiology, where metal-resistant microorganisms colonize contaminated soils and industrial environments exposed to heavy metals (Bruins et al., 2000; Nies, 2003). Within the human gut, members of the Lactobacillales, Bacillaceae, and Clostridiaceae have been identified among the taxa most frequently associated with nickel resistance, whereas Enterobacteriaceae tend to decrease progressively as nickel concentrations increase (Lusi et al., 2017).

To provide a concise overview of the principal microbial groups currently associated with nickel resistance, their putative resistance mechanisms, their potential ecological and physiological significance, a summary is presented in Table 1.

**Table 1:** Nickel-resistant bacterial taxa identified in the human gut microbiome and their potential ecological and physiological significance.

Bacterial taxon/group	Observation under nickel exposure	Putative nickel-resistance mechanisms	Potential ecological significance	Potential implications for host physiology
Lactobacillales (Lactobacillaceae, Enterococcaceae, Streptococcaceae)	Progressive enrichment at increasing nickel concentrations; particularly abundant in overweight nickel-allergic women	Cell wall adsorption of metal ions, extracellular sequestration, stress-response pathways, metal binding	Ecological adaptation to metal-rich environments; competitive advantage under nickel selection pressure	Reduced nickel bioavailability, modulation of immune responses, altered microbial metabolite production
Bacillaceae (Bacillales)	Increased abundance at extreme nickel concentrations	Metal efflux pumps, intracellular sequestration, metallo-regulatory systems	Survival under conditions of elevated metal stress; ecological restructuring of microbial communities	Potential contribution to gut microbial resilience and adaptation to chronic nickel exposure
Clostridiaceae (Clostridiales)	Increased abundance in highly nickel-tolerant microbial communities	Metal tolerance pathways, detoxification systems, stress adaptation mechanisms	Expansion of anaerobic populations adapted to metal-rich conditions	Potential influence on fermentation pathways, short-chain fatty acid production, and host-microbiome interactions
Enterobacteriaceae (Enterobacteriales)	Progressive reduction with increasing nickel concentrations	Lower tolerance to elevated nickel levels compared with resistant taxa	Loss of nickel-sensitive populations and reduced community diversity	Marker of nickel-driven ecological selection within the gut microbiota
Nickel-resistant gut microbiota (overall community)	Growth at nickel concentrations substantially exceeding those tolerated by microbiota from non-allergic controls	Combined ecological adaptation involving metal transport, efflux, sequestration and stress-response systems	Emergence of a microbial community adapted to chronic environmental nickel exposure	Candidate biological interface linking dietary nickel exposure, immunity, microbial ecology and metabolic homeostasis

Although the biological significance of these microbial changes remains incompletely understood, the emergence of nickel-resistant communities raises important questions regarding the relationship between environmental metal exposure, microbial adaptation, host immunity, and metabolic health. Whether these microorganisms merely represent biomarkers of chronic nickel exposure or actively participate in metabolic regulation remains one of the central unresolved questions in the field. Future studies integrating microbiology, metabolomics, immunology, and experimental models will be required to clarify their role and biological significance.

## Mechanistic interplay: detoxification versus metabolic modulation

The emergence of nickel-resistant microbiota introduces an intriguing biological framework in which microbial adaptation to environmental metal exposure may have consequences extending beyond detoxification. Microorganisms have evolved multiple mechanisms to tolerate elevated nickel concentrations, including metal efflux pumps, intracellular sequestration systems, metallo-regulatory proteins, metallo-chaperones, and biofilm-associated resistance pathways (Bruins et al., 2000; Nies, 2003). These adaptive strategies have been extensively described in environmental microbiology and are likely to contribute to the persistence of nickel-resistant bacterial populations within the gastrointestinal tract.

Among the microbial groups enriched under increasing nickel concentrations, Lactobacillales have been reported to bind metal ions through cell wall components and extracellular polymers, potentially reducing luminal nickel bioavailability and limiting host exposure. Likewise, members of the Bacillaceae and Clostridiaceae possess metal-responsive regulatory systems and resistance mechanisms that may facilitate survival in nickel-rich environments (Bruins et al., 2000; Nies, 2003). Collectively, these observations suggest that nickel-resistant microorganisms may represent adaptive responses to chronic dietary metal exposure.

Beyond detoxification, microbial adaptation may also influence host physiology. Gut microorganisms are known to participate in the production of short-chain fatty acids, bile acid transformation, amino acid metabolism, and immune signaling pathways, all of which contribute to metabolic homeostasis (Chen and Devaraj, 2018). It is therefore plausible that chronic ecological selection of nickel-resistant communities could alter microbial metabolic outputs and consequently influence host metabolic responses.

However, direct evidence linking nickel-resistant microbiota to metabolic dysfunction remains limited. At present, it is unclear whether these microbial populations actively contribute to metabolic alterations, represent adaptive responses to prolonged nickel exposure, or simply serve as biomarkers of environmental metal burden. Alternative explanations, including dietary habits, host genetics, immune status, and obesity-associated physiological changes, may also contribute to the observed associations.

Nickel-driven microbial shifts may further influence host immunity. Alterations in microbial composition and metabolite production have the potential to affect gut barrier function and inflammatory signaling pathways, both of which are implicated in obesity and metabolic syndrome. Nevertheless, the precise contribution of nickel-resistant microbial communities to these processes remains to be determined.

Taken together, current evidence supports the hypothesis that nickel-resistant microbiota may constitute a candidate biological interface linking environmental nickel exposure, microbial adaptation, immune responses, and metabolic health. Future studies integrating metagenomics, metabolomics, controlled dietary interventions, and microbiota-transfer experiments will be necessary to determine whether these microbial communities are merely markers of exposure or active participants in disease pathogenesis.

## Extending the framework: a one health perspective

The potential implications of the nickel–gut microbiome axis extend beyond human health and support consideration within a broader One Health framework, which recognizes the interconnectedness of environmental, animal and human health. Nickel is a ubiquitous environmental contaminant present in soil, water, vegetation and agricultural systems, creating continuous opportunities for exposure across multiple species and ecological niches.

Microorganisms exposed to chronic nickel contamination have long been recognized in environmental microbiology, where metal-resistant populations emerge as adaptive responses to selective pressure (Bruins et al., 2000; Nies, 2003). Similar processes may occur within animal gastrointestinal ecosystems. Livestock, companion animals, wildlife and aquatic organisms are routinely exposed to nickel through feed, water, and environmental sources, potentially promoting the selection of nickel-resistant microbial communities analogous to those described in humans.

The biological consequences of these adaptations remain largely unexplored. Nickel-resistant taxa such as Lactobacillales, Bacillaceae, and Clostridiaceae may influence nutrient utilization, microbial ecology, immune responses, and metabolic homeostasis across species. Understanding whether these microbial shifts represent beneficial adaptive responses, biomarkers of chronic exposure, or contributors to metabolic dysfunction is therefore relevant not only to human medicine but also to veterinary medicine, animal production, and environmental health.

Importantly, environmental metal contamination does not affect individual species in isolation. Through shared ecosystems, food chains and water sources, nickel exposure may influence microbial communities across environmental, animal and human compartments. This raises the possibility that nickel-driven microbial selection represents a broader ecological phenomenon with implications extending beyond a single host species.

Experimental animal models offer a valuable opportunity to investigate these questions under controlled conditions. Zebrafish (*Danio rerio*) provide a tractable model for examining the effects of dietary nickel exposure on microbial composition, inflammation, and metabolic outcomes, whereas rodent models could be used to evaluate the functional consequences of nickel-resistant microbiota through microbiota-transfer experiments, controlled dietary interventions, and integrated metabolomic analyses. Such approaches may help clarify whether nickel-resistant microbial communities actively influence host physiology or simply reflect adaptation to environmental metal exposure.

From a One Health perspective, the study of nickel-resistant microbiota may also provide insight into broader ecological processes linking environmental contamination, microbial adaptation, and host health. Future comparative investigations across human, animal, and environmental systems may reveal whether nickel-driven microbial selection represents a conserved biological response to chronic metal exposure and whether similar mechanisms contribute to metabolic and inflammatory disorders across species.

## Conclusion and implications for future research

The recognition that chronic dietary nickel exposure is associated with overweight and metabolic alterations in susceptible individuals has broadened the traditional view of nickel as a mere allergen and opened a new area of investigation at the intersection of environmental exposure, immunity, microbiology, and metabolism (Lusi et al., 2015; Watanabe et al., 2018). The subsequent identification of nickel-resistant bacteria within the human gut microbiota (Lusi et al., 2017) added a new biological dimension to this emerging field, suggesting that microbial adaptation may represent one pathway through which environmental nickel exposure interacts with host physiology.

Taken together, the available evidence supports the hypothesis that chronic nickel exposure may act not only as an allergen but also as an ecological selective pressure capable of shaping gut microbial communities. Although causality remains to be established, the proposed nickel–gut microbiome axis provides a plausible conceptual framework for investigating the complex interactions among environmental exposure, microbial ecology, immune responses, and metabolic health.

Future research integrating microbiology, immunology, metabolism, and environmental sciences will be essential to clarify these relationships. Longitudinal human studies, controlled dietary interventions, experimental animal models, microbiota-transfer approaches, and multi-omics investigations will be required to determine whether nickel-resistant microbial communities are biomarkers of exposure, adaptive responses, or active contributors to disease pathogenesis.

By identifying key knowledge gaps and proposing testable hypotheses, this Perspective aims to stimulate further investigation into a developing field whose biological and clinical significance remains incompletely understood. Ultimately, a deeper understanding of the interactions between nickel exposure, gut microbiota, and host physiology may contribute to novel strategies for the prevention and management of metabolic disorders in susceptible individuals.

## Data Availability

The original contributions presented in the study are included in the article/supplementary material, further inquiries can be directed to the corresponding author.

## References

[ref1] Bruins M. R. Kapil S. Oehme F. W. (2000). Microbial resistance to metals in the environment. Ecotoxicol. Environ. Saf. 45, 198–207. doi: 10.1006/eesa.1999.1860, 10702338

[ref2] Chen X. Devaraj S. (2018). Gut microbiome in obesity, metabolic syndrome, and diabetes. Curr. Diab. Rep. 18:129. doi: 10.1007/s11892-018-1104-3, 30338410

[ref3] Lusi E. A. Di Ciommo V. M. Patrissi T. Guarascio P. (2015). High prevalence of nickel allergy in an overweight female population: a pilot observational analysis. PLoS One 10:e0123265. doi: 10.1371/journal.pone.0123265, 25822975 PMC4379055

[ref4] Lusi E. A. Patrissi T. Guarascio P. (2017). Nickel-resistant bacteria isolated in human microbiome. New Microbes New Infect. 19, 67–70. doi: 10.1016/j.nmni.2017.06.001 28725438 PMC5501881

[ref5] Nies D. H. (2003). Efflux-mediated heavy metal resistance in prokaryotes. FEMS Microbiol. Rev. 27, 313–339. doi: 10.1016/S0168-6445(03)00048-2, 12829273

[ref6] Rehman K. Fatima F. Waheed I. Akash M. S. H. (2018). Prevalence of exposure of heavy metals and their impact on health consequences. J. Cell. Biochem. 119, 157–184. doi: 10.1002/jcb.26234, 28643849

[ref7] Watanabe M. Masieri S. Costantini D. Tozzi R. De Giorgi F. Gangitano E. . (2018). Overweight and obese patients with nickel allergy have a worse metabolic profile compared to weight matched non-allergic individuals. PLoS One 13:e0202683. doi: 10.1371/journal.pone.0202683 30153310 PMC6112671

[ref8] Zambelli B. Uversky V. N. Ciurli S. (2016). Nickel impact on human health: an intrinsic disorder perspective. Biochim. Biophys. Acta 1864, 1714–1731. doi: 10.1016/j.bbapap.2016.09.008, 27645710

